# A Muscle Energy Techniques-Based Physiotherapeutic Intervention Protocol for Managing Nightstick Fracture: A Case Report

**DOI:** 10.7759/cureus.53353

**Published:** 2024-01-31

**Authors:** Aditi P Ambekar, Mitushi Deshmukh, Vaishnavi M Thakre, Pooja S Ladkhedkar, Palak R Ahuja

**Affiliations:** 1 Musculoskeletal Physiotherapy, Ravi Nair Physiotherapy College, Datta Meghe Institute of Higher Education and Research, Wardha, IND

**Keywords:** ulna, fracture, malunion, radius, motion, supination, pronation

## Abstract

Distal ulna and radius fractures are the most frequent upper extremity fractures seen in emergency rooms. The axis of rotation for forearm pronation and supination runs through the radial head (proximal) and the ulnar fovea (distal). Throughout pronation and supination, the radius can rotate relative to the ulna, thanks to the way its head articulates with it. The ulna remains relatively stable during these movements. However, in cases of fractures of these bones, surgery to repair the radius is usually the best course of action for a distal ulna fracture. Most distal ulna fractures heal successfully with only conservative treatment once the radius is stabilized. To achieve the best results, medical personnel must take into account patient characteristics including age, level of activity, and aspirations. The majority of distal ulna injuries do not require surgery, but there are several circumstances where it is necessary. In therapeutic practice, muscle energy techniques (METs) are comparatively painless methods for restoring a restricted spectrum of motion. Malunion, reduced grasp, and other significant problems might result from a lack of understanding of this illness. The 48-year-old patient in the present study was reported to have sustained injuries to his left forearm in a road traffic accident (RTA) as he fell from his bike and slid during a traffic collision. X-ray imaging of the left forearm revealed an isolated ulnar shaft fracture. METs, isometric contractions, and active concentric and eccentric movements were all part of the physiotherapy intervention protocol to produce an active range of motion in the upper extremity. In this particular case, the specified physiotherapy management was found to be effective.

## Introduction

The interplay between the radius and ulna creates a biomechanical balance that is crucial for the range of motion in the forearm. The combination of pronation and supination allows for a complete 180-degree arc of motion of the hand. The curved structure of the radius facilitates rotation around the stable ulna, playing a crucial role in this movement. Any disturbance in the forearm's anatomy could result in a notable reduction in the usual range of motion. Rare forearm fractures referred to as isolated ulnar shaft fractures may result after a fall or an immediate blow to the forearm. Often these fractures are termed as "nightstick" fractures [1]. Plain radiography (X-rays) and physical examination are the main diagnostic tools. The incidence of upper extremity fractures is considered rare. They account for less than 1% of all fractures [2]. The highest incidence of upper extremity fractures is observed in two distinct demographic groups: men between the ages of 10 and 20 years and women over the age of 60 years [3,4]. For instance, a "nightstick" fracture occurs when there's a direct blow to the forearm. This can result in an isolated fracture of the ulna, usually transverse and located in the mid-diaphysis [5]. Automobile collisions, altitude tumbles, and axial loads transmitted via the hand to the forearm are examples of mechanisms that result in indirect trauma. These mechanisms cause injury by applying forces away from the site of the fracture [6]. In stable fractures of the ulna, displacement is less than 50%; this means that the broken bone segments have not shifted significantly from their original positions [7]. If the displacement is minimal (less than 50%), it suggests that the fracture is relatively stable [8]. Also, the periosteum remains intact, which can help keep the bone fragments in place. The interosseous membrane is intact and acts as a restraint to rotation; it contributes to the stability of the fracture [9].

The periosteum and interosseous membrane are affected together with certain related complications like a radial head injury or displacements, and these are the hallmarks of an unstable fracture, especially in the setting of a forearm injury [10]. A physiotherapy approach was put together to increase the upper limb's active range of motion, avoid contractures, and lessen muscle stiffness. To improve muscular activity and expand the limited range of motion, muscle energy techniques (METs), isometric contractions, and active concentric and eccentric motions were used in tandem. We devised an exercise program centered on MET to assist patients dealing with fracture instances in enhancing their mobility, freedom in daily activities, and minimizing problems.

## Case presentation

Patient information

We present a case of a 48-year-old male patient who fell from his bike and slid during a traffic collision at around 5:30 p.m. on September 22, 2023 and came to Acharya Vinoba Bhave Rural Hospital (AVBRH) casualty department sustaining an injury to his left forearm. The patient had undergone certain investigations, including an X-ray of the left forearm and chest radiography, and was diagnosed with an isolated mid-shaft ulnar fracture. The patient complained of severe pain in the left forearm, which was acute in onset and gradual in progression. It aggravated on movement and relieved with rest. A below-elbow slap was applied to the left forearm. For additional care, the patient received a referral to the orthopedic department. Open reduction and internal fixation (ORIF) with plate osteosynthesis (seven-hole limited contact dynamic compression plate and six screws) under locoregional anesthesia was performed on September 24, 2023. The patient experienced pain following surgery, and there was limited mobility and motion across the elbow and wrist joint. On September 27, 2023, physiotherapy was commenced with a proper protocol for the patient.

Clinical findings

Before the commencement of the examination, the patient gave his informed consent and then underwent evaluation. On examination, the patient was afebrile and hemodynamically stable. The individual was observed resting recumbent with his head end raised to a 30° angle. The left forearm was cast and hanging in a triangular sling. His vision and hearing were normal. During a neurological evaluation, sensations were intact. The tone and muscle strength were reduced in the upper limbs. The mobility was reduced in the upper limb (Table 1). All deep tendon reflexes were normal. The patient was unable to stand and walk. As evaluated by the Functional Independence Measure (FIM), the patient required the highest level of help for both instrumental and basic daily living tasks, such as dining, washing, moving, and using the restroom (transport, interaction, and medicine management).

**Table 1:** Pre-intervention range of motion of the upper limb

Upper limb - Joints	Right	Left
Shoulder - Flexion	0^o^-160^o^	0^o^-50^o^
Extension	0^o^-50^o^	0^o^-10^o^
Abduction	0^o^-160^o^	0^o^-120^o^
Adduction	0^o^	0^o^
Internal rotation	0^o^-50^o^	0^o^-50^o^
External rotation	0^o^-50^o^	0^o^-20^o^
Elbow - Flexion	0^o^-140^o^	0^o^-60^o^
Extension	140^o^-0^o^	60^o^-0^o^
Forearm - Supination	0^o^-70^o^	0^o^-30^o^
Pronation	0^o^-70^o^	0^o^-30^o^
Wrist - Flexion	0^o^-80^o^	0^o^-40^o^
Extension	0^o^-80^o^	0^o^-40^o^
Ulnar deviation	0^o^-20^o^	0^o^-10^o^
Radial deviation	0^o^-40^o^	0^o^-20^o^

Diagnostic assessment

**Figure 1:**
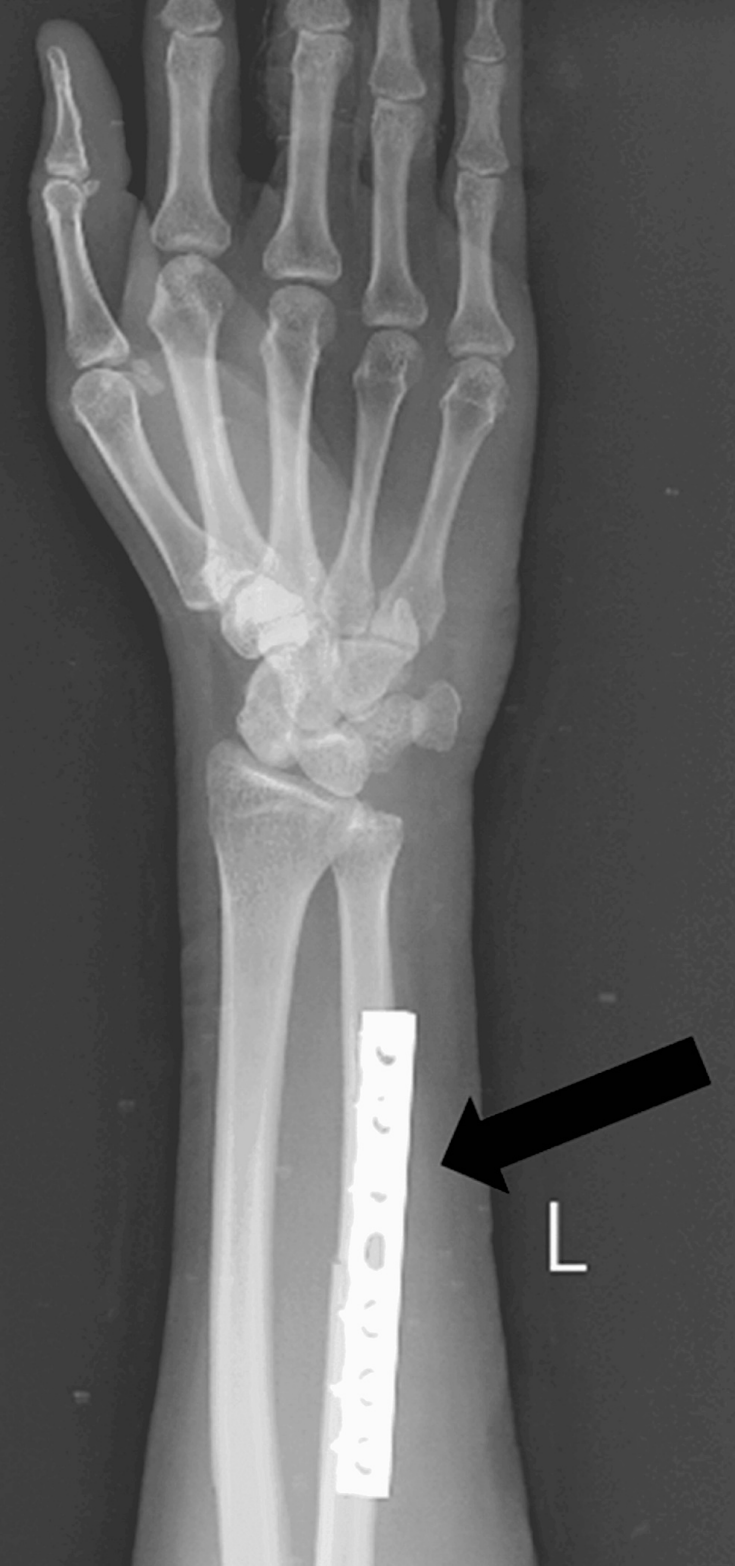
Post-operative X-ray of the left forearm The arrow represents an isolated ulnar shaft fracture managed by open reduction and internal fixation with plate osteosynthesis.

An investigation of the cerebrospinal fluid (CSF) and a complete blood count (CBC) were performed. An isolated ulnar shaft fracture being treated with ORIF with plate osteosynthesis (seven-hole limited contact dynamic compression plate and six screws) is shown in the left forearm X-ray (Figure 1).

Timeline of the current episode

In September 2023, the patient was admitted to the orthopedic ward after the surgery. He was recommended for a physiotherapeutic regimen on day three. The patient underwent three weeks of therapy.

Physiotherapy intervention 

**Figure 2:**
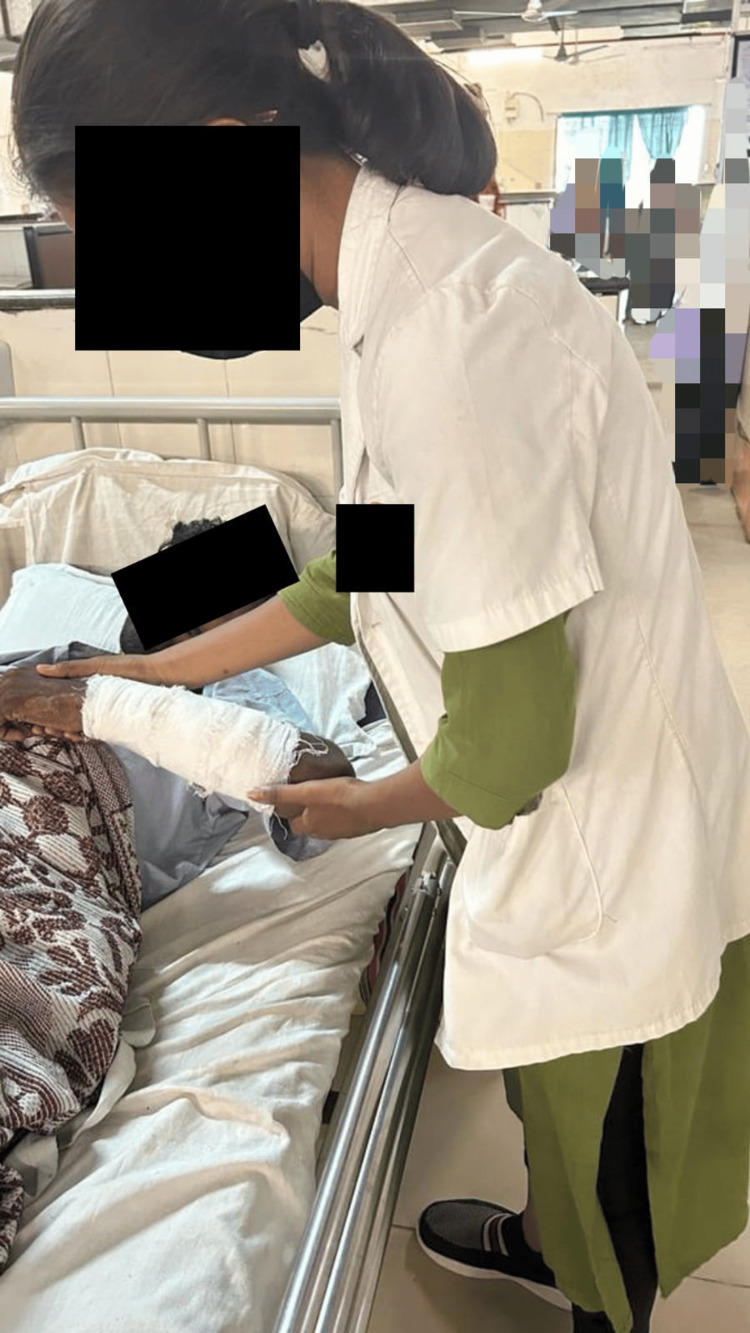
Patient undergoing MET for wrist extensors MET: Muscle energy technique

**Figure 3:**
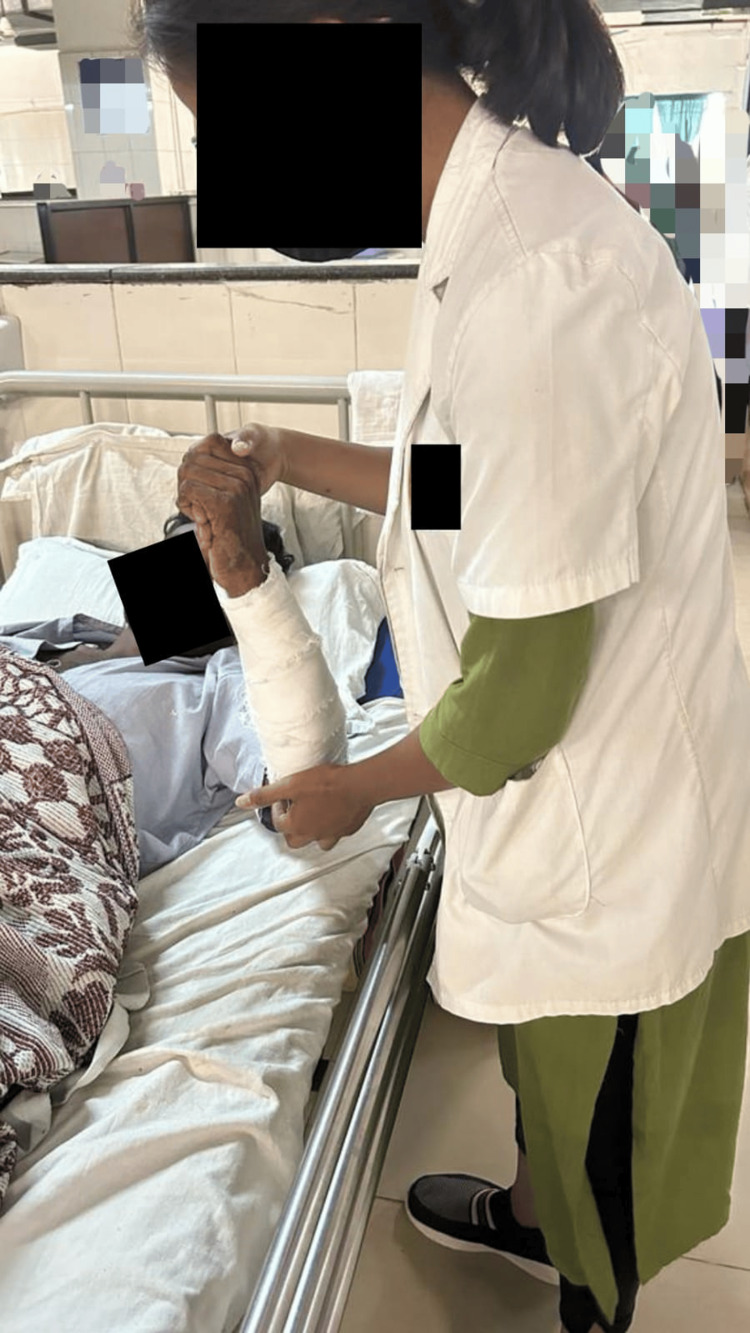
Patient undergoing MET for wrist flexors MET: Muscle energy technique

Physiotherapy intervention protocol to achieve an active range of motion of the upper limb had been established to reduce the tightness of muscles and prevent contractures. In the initial phases of post-fracture recuperation, when open reduction and rigid internal fixation are utilized surgically, MET can be given effectively as an adjuvant to the physical therapy program to address forearm pain. It is an array of mobilization techniques used to restore function, reduce tissue edema, relieve muscular spasms, extend the integrity of the tissue, and teach stabilizing skills of the intersegmental linked musculature. In this procedure, the individual precisely controls and consciously contracts the muscle against the therapist's counterforce. Table 2 and Figures 2-3 illustrate the METs used to restore the functions and mobility of the elbow and forearm. To regain range of motion, a combination of active concentric and eccentric exercises and isometric contractions were used.

**Table 2:** Physiotherapy intervention MET: Muscle energy technique; NA: Not applicable; Reps: Repetition; ROM: Range of motion

Physiotherapy interventions	Type of contraction and muscle group	Duration and repetitions
MET: Post-isometric relaxation (PIR)	Submaximal (10-20%) contraction of elbow flexors & extensors	For 5-10 seconds; resistance is offered in the opposite direction; Reps - 2 or 3 times
MET: Post-facilitation stretching (PFS)	Elbow flexors to their utmost potential in order to elongate the agonist	For 5-10 seconds while the therapist opposes the force used by the patient; Reps - 3 to 5 times
MET: Reciprocal inhibition	Isometric & isotonic contractions of supinator and pronators	For 5-10 seconds; Reps - 3 to 5 times
Cryotherapy for pain reduction	NA	For 7 minutes daily
Gripping exercises	NA	10 repetitions × 1 set; For 3 weeks
Active ROM exercises	NA	10 repetitions × 1 set; For 3 weeks
Strengthening exercises for elbow using half litre bottle	NA	10 repetitions × 1 set; For 3 weeks

Follow-up and outcome measures

An integrative regimen for physical rehabilitation was established. The subsequent investigation was conducted once a week for three weeks. The outcome measure results are displayed in Tables 3-4.

**Table 3:** Post-intervention range of motion of the upper limb

Upper limb - Joints	Right	Left
Shoulder - Flexion	0^o^-160^o^	0^o ^-120^o^
Extension	0^o^-50^o^	0^o^-60^o^
Abduction	0^o^-160^o^	0^o^-120^o^
Adduction	0^o^	0^o^
Internal rotation	0^o^-50^o^	0^o^-60^o^
External rotation	0^o^-50^o^	0^o^-60^o^
Elbow - Flexion	0^o^-140^o^	0^o^-110^o^
Extension	140^o^-0^o^	110^o^-0^o^
Forearm - Supination	0^o^-70^o^	0^o^-50^o^
Pronation	0^o^-70^o^	0^o^-50^o^
Wrist - Flexion	0^o^-80^o^	0^o^-80^o^
Extension	0^o^-80^o^	0^o^-80^o^
Ulnar deviation	0^o^-20^o^	0^o^-20^o^
Radial deviation	0^o^-40^o^	0^o^-30^o^

**Table 4:** Outcome measures pre- and post-rehabilitation DASH: Disabilities of the arm, shoulder, and hand; DASH scale: 0 represents no disability, 100 represents severe disability

Scales	Pre-treatment score	Post-treatment score
DASH scale	40/100	10/100
Functional Independence Measure	16/126	45/126

## Discussion

The primary goal of upper limb care during the acute phase is to avoid secondary problems such as edema and contractures. According to research by Lalwani et al., early mobility, adequate lower limb strength, pain management, and quality of life are all important factors. The intertrochanteric fracture rehabilitation program is helpful, as shown by statistically significant increases in exercise ability and well-being [11,12]. Therapies available for isolated minor or non-displaced ulnar shaft fractures include functional bracing or casting, attentive monitoring, and periodic evaluations [13]. The distal radioulnar (DRU) joint's stability over the long term must be taken into account when addressing acute ulnar styloid fractures [14]. It is decided by the relationship between the ulnar styloid and the stabilizing ligaments if a particular type of injury has the potential to create instability in the DRU joint [15]. Primary reduction and immobilization need to be done to optimally prep the patient for surgical procedures since the majority of forearm fractures necessitate surgery [16]. To increase elbow extension, METs are administered [17]. These are patient-directed approaches that result in faster, more favorable outcomes [18]. According to some research, a hybrid fixation approach is used because it offers superior stability, fewer difficulties, and positive clinical outcomes by combining plate fixation of the radius with intramedullary nail placement of the ulna [19]. During rehabilitation, physical therapy plays a critical role in dealing with elbow extensor lagging [20]. The study conducted by Chandak et al. has determined that the administration of METs is effective in augmenting elbow extension [21]. MET interventions represent immediate therapeutic measures that yield favorable outcomes. Furthermore, the demonstrated efficacy of this procedure suggests its potential incorporation into future therapeutic management strategies. Specifically, the identified benefits include the resolution of extensor lag of the elbow and the mitigation of post-fracture elbow stiffness. In addition, the investigation conducted by Faqih et al. suggests that METs have served as a supplementary intervention within the rehabilitation protocol for addressing elbow stiffness [22]. Furthermore, it is deemed safe to administer METs during the initial phases of post-operative elbow fracture rehabilitation, particularly in cases managed surgically through open reduction and rigid internal fixation. In this case, cryotherapy was administered pre-exercise to manage post-operative pain and alleviate discomfort. Concurrently, the patient underwent analgesic drug therapy for pain management. The integration of METs alongside isometric, concentric, and eccentric exercises demonstrated efficacy in facilitating the attainment of an active range of motion for the elbow, forearm, and wrist joints in this patient.

## Conclusions

In this case, METs are targeted to specific musculature of the hand and forearm, contributing to relaxation, tension relief, prevention of muscle imbalances, and facilitation of a more streamlined recovery process. METs demonstrate efficacy in rectifying specific imbalances among forearm muscles, fostering symmetry, and optimizing the functionality of the elbow, forearm, and wrist. Preceding physiotherapeutic interventions, cryotherapy was employed to manage post-operative pain. METs entail active patient involvement, resembling an "active release" technique. This approach incorporates controlled muscle contractions and relaxation to reset muscle length and tone, addressing hypertonicity and augmenting joint function. In addition to the METs, the physiotherapy intervention encompasses isometric contractions, as well as active concentric and eccentric movements. The recommended course of physiotherapy for the restoration of functional ranges and strength involves an extended follow-up period and intensive therapy spanning three to four weeks, followed by comprehensive patient education.
